# Investigating the role of electroanatomical mapping in single‐shot pulsed field catheter ablation

**DOI:** 10.1002/joa3.13180

**Published:** 2024-11-10

**Authors:** Ourania Kariki, Panagiotis Mililis, Athanasios Saplaouras, Theodoros Efremidis, Anastasios Chatziantoniou, Ioannis Panagiotopoulos, Stylianos Dragasis, Konstantinos P. Letsas, Michael Efremidis

**Affiliations:** ^1^ Arrhythmia Unit Onassis Cardiac Surgery Center Athens Greece

**Keywords:** atrial fibrillation, catheter ablation, electroanatomical mapping, pulmonary vein isolation, pulsed field ablation

## Abstract

**Introduction:**

Pulsed field ablation (PFA) is a form of nonthermal energy that has been recently introduced for pulmonary vein isolation (PVI). A multi‐electrode pentaspline catheter for delivery of PFA guided by fluoroscopy has become widely available for clinical use.

**Methods and Results:**

In this study, we aimed to assess whether the addition of electroanatomical mapping (EAM) for confirmation of PVI in the acute phase can increase the efficacy of the procedure in terms of arrhythmia recurrences. A total of 51 patients with atrial fibrillation (AF) scheduled for first time PVI were included in the study. Participants were assigned to receive PVI using fluoroscopy guidance only (Fluoro‐only group: 31 patients) or additional validation with EAM (EAM group: 20 patients). Endpoints included arrhythmia recurrence and procedural characteristics. During a 11.2 ± 1.3 months follow‐up period, arrhythmia recurrences did not statistically differ between groups (16.1% vs. 20%, *p* .72). Procedure time was longer in the EAM group (86.5 ± 11.4 vs. 78.4 ± 9.3 min, *p* .008). EAM revealed 5 nonisolated PVs that were re‐ablated using the same catheter. Four patients of the cohort underwent a redo‐procedure during the follow‐up period. In all 4 cases, at least one reconnected PV was identified.

**Conclusion:**

In a cohort of patients with AF undergoing first time PVI using a pentaspline PFA catheter, PVI validation with EAM did not lead to significantly different arrhythmia recurrence rates compared to PVI without EAM. In the acute phase, the rate of nonisolated PVs was low.

## Graphical abstract

In a cohort of patients undergoing first time pulmonary vein isolation with the pentaspline pulsed‐field ablation catheter, validation in the acute phase of successful pulmonary vein isolation by electroanatomical mapping did not lead to significantly different arrhythmia recurrence rates.

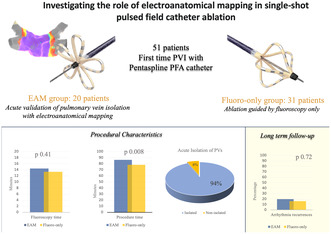

## 1 INTRODUCTION

Pulmonary vein isolation (PVI) with wide antral lesions in the pulmonary vein (PV) ostium is the cornerstone of all ablation techniques for atrial fibrillation (AF). Pulsed field ablation (PFA) is a novel form of nonthermal energy in electrophysiology, demonstrating enhanced safety properties for PVI.^1^ Delivery of PFA lesions using a multi‐electrode pentaspline catheter, guided by fluoroscopy, was the first commercially available device incorporating PFA energy.^2^ The nonuse of three‐dimensional electroanatomical mapping (EAM) for guidance is not an uncommon practice for single shot devices that rely mainly on anatomical manipulation of the catheter for the PVI. The unique design of the catheter in combination with the lack of clinical experience of the characteristics of PFA‐induced myocardial lesions inevitably requires a significant number of real‐world studies to provide insights regarding the most efficient ways to apply this novel technology. The aim of the current study was to assess whether the addition of EAM to confirm successful PVI after ablation with a multi‐electrode pentaspline PFA catheter can enhance the long‐term rhythm control efficacy of the procedure.

## 2 MATERIALS AND METHODS

### 2.1 Study design and patient selection

This is a prospective, nonrandomized, single‐center study. Inclusion criteria were: age >18 years, first time PVI using the FARAPULSE™ PFA System (Boston Scientific, Natick, MA, USA) and willingness to participate in the study. Exclusion criteria were previous intervention for AF (transcatheter ablation or surgery), intracardiac thrombus, contraindication to anticoagulation, and more than moderate valvular disease. Patients were separated in two groups. The first group received PVI using fluoroscopy only (Fluoro‐only). The second group, after the completion of PVI, received supplementary EAM to validate the result. In cases of unsuccessful isolation, additional lesions were delivered with the pentaspline catheter and remapping was performed (EAM group). Primary endpoint of the study was arrhythmia recurrence (any atrial tachyarrhythmia (ATA) episode lasting more than 30 s) in a 12‐month follow‐up. Secondary endpoints included procedural characteristics (procedure time, fluoroscopy time, nonisolated PVs identified by the EAM in the acute phase). The study was approved by institutional board and ethical committee. The enrolment period lasted from February to July 2023.

### 2.2 Procedure workflow

All patients provided written informed consent before the procedures. Antiarrhythmic drugs were discontinued for at least five half‐lives prior to procedures while all patients received anticoagulation therapy for at least 3 weeks. Procedures were performed under general anesthesia with intubation. After two femoral vein punctures, a steerable decapolar catheter was used for the catheterization of the coronary sinus. Left atrium was accessed through a single transseptal puncture with a transseptal guiding introducer sheath and a transseptal needle under fluoroscopy. Transesophageal guidance or intracardiac echocardiography was used whenever needed. Heparin was administered prior to the transseptal puncture and the active clotting time was maintained above 350 s throughout the procedure. After a successful transseptal puncture, the transseptal sheath was exchanged for a 13‐F steerable sheath (FARADRIVE™). A 12‐F over‐the‐wire ablation catheter (FARAWAVE™) was then introduced in the left atrium guided by a J‐tip guidewire of 0.035‐inch diameter. Following PV cannulation with the wire, ablation was performed with four applications in basket and six applications in flower configuration for each PV (two extra applications in flower configuration with anterior torque). To cover the entire circumference, the catheter was slightly rotated by 30–40° after the first two applications in each configuration. PVI was defined as elimination of PV potentials at the PV ostium with the ablation catheter in a basket position and establishment of entrance and exit block with pacing maneuvers. In the cases of nonisolated PV, additional applications were performed until isolation.

For patients assigned to receive validation mapping with EAM, the ablation catheter was exchanged for a multipolar diagnostic catheter [Advisor™ HD Grid catheter (Abbott Laboratories, Abbott Park, Ill, USA)]. EAM of the left atrium was performed using the EnSite Precision™ cardiac mapping system (Abbott, St. Paul, Min). PVI was defined as low voltage at the PV antrum with establishment of entrance and exit block with pacing maneuvers. In cases of nonisolated PVs, additional lesions were delivered with the ablation catheter and remap was performed to confirm isolation.

### 2.3 Follow‐up

Patients with uncomplicated procedures were discharged the next day. Follow‐up included 24‐h Holter monitoring in 1, 3, 6, 9, and 12 months with additional electrograms in case of symptoms.

### 2.4 Statistical analysis

Categorical variables were tested using the Chi‐square and Fisher's exact test as appropriate. Continuous variables were tested for normality using Kolmogorov–Smirnov test and compared using Student *t* test and the Mann–Whitney *U* test, as appropriate. The significance level was defined as *p* < .05 for two‐sided tests. The SPSS statistical package (version 26.0; SPSS Inc., Chicago, IL) was used.

## 3 RESULTS

A total of 51 patients were included in the study (20 in EAM and 31 in Fluoro‐only group). The mean age of the cohort was 62.4 ± 10.4 years and 21 (41.2%) were males. The majority of the patients of the cohort had paroxysmal type of AF (42/51) and the differences in AF type were not statistically significant between groups (14 patients with paroxysmal AF in EAM group vs. 28 in Fluoro‐only group, *p* .06). The mean follow‐up was 11.2 ± 1.3 months. Patient characteristics were comparable between groups. Baseline demographics are shown in Table 1.

**TABLE 1:** Patients' characteristics.

	Total patients, *n* = 51	EAM group, *n* = 20	Fluoro‐only group, *n* = 31	*p*‐value
Clinical characteristics				
Age, years	62.4 ± 10.4	59.5 ± 12.7	64.3 ± 8.4	.11
Males	21 (41.2%)	9 (45%)	14 (45.2%)	.99
Height	170.2 ± 11	172.6 ± 11.4	168.7 ± 10.7	.23
Weight	85.5 ± 19.1	90.7 ± 22.7	82 ± 15.8	.12
CHA_2_DS_2_‐VA score	1.4 ± 1	1.5 ± 1	1.3 ± 1.1	.49
HASBLED score	0.6 ± 0.61	0.6 ± 0.7	0.6 ± 0.6	.91
Paroxysmal AF	42 (82.4%)	14 (70%)	28 (90.3%)	.06
Echocardiography				
LVEF, %	58.9 ± 2.7	59.3 ± 1.8	58.7 ± 3.2	.49
LA diameter, mm	38.7 ± 4.7	39 ± 5.4	38.6 ± 4.3	.79

### 3.1 Procedural characteristics

**FIGURE 1:**
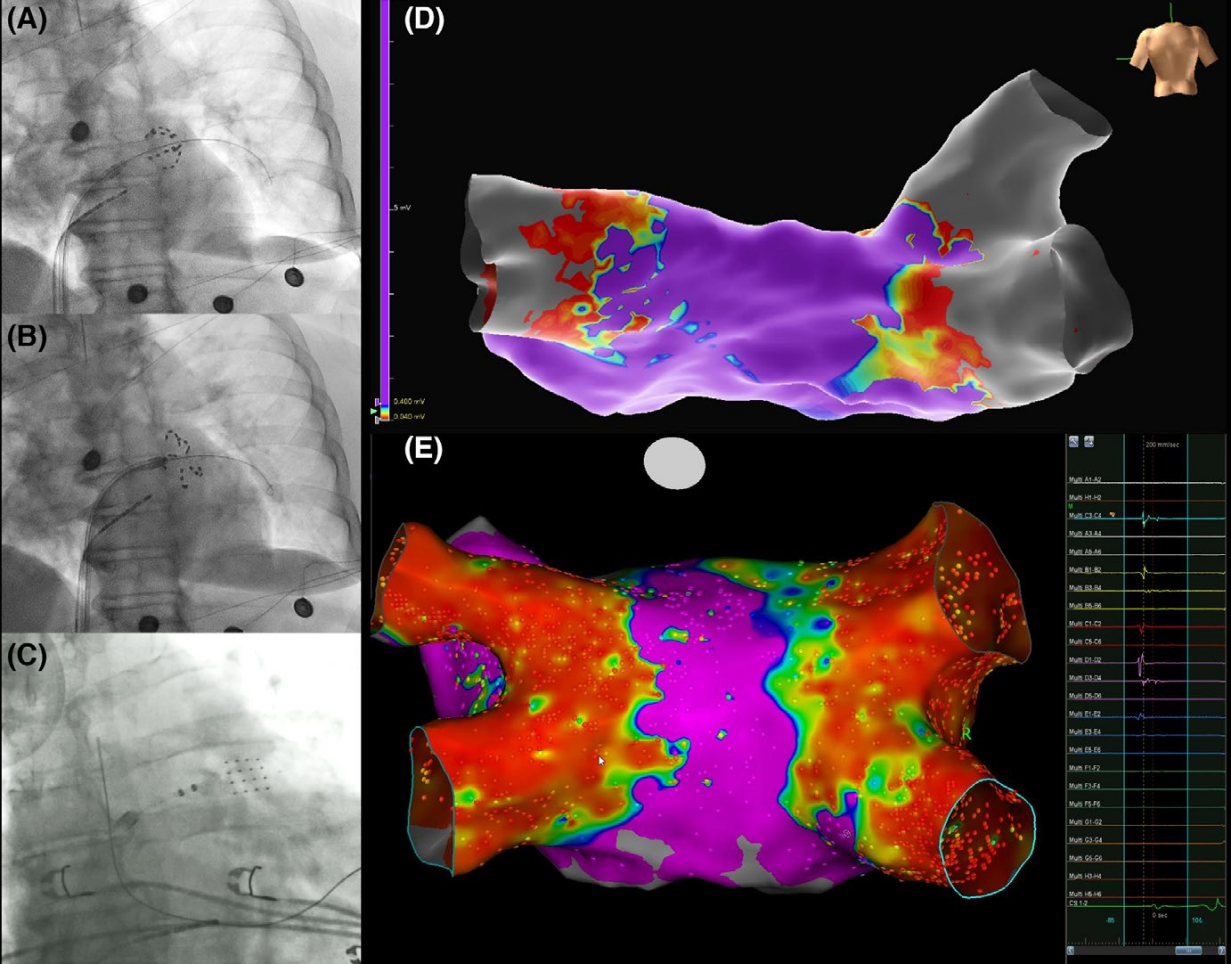
(A, B) The pentaspline catheter in basket and flower configuration during the isolation of the left inferior pulmonary vein, (C) Exchange of the ablation catheter with a multipolar diagnostic catheter for validation of pulmonary vein isolation using electroanatomical mapping, in the EAM group, (D) Three‐dimensional model of the left atrium of a patient with common ostium of the left pulmonary veins. Successful isolation was confirmed. (E) A case of redo procedure with reconnection of the right inferior pulmonary vein.

**FIGURE 2:**
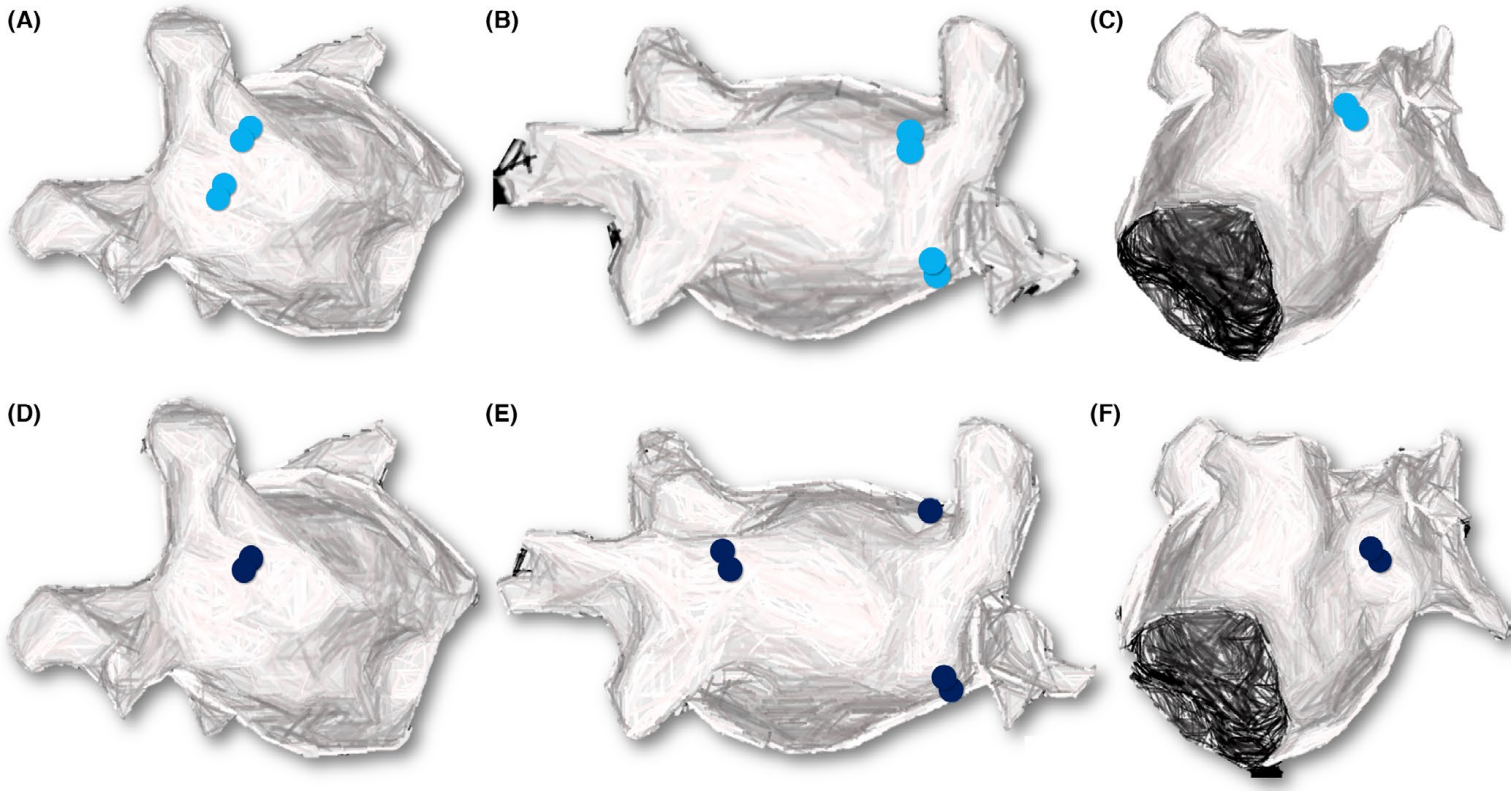
Sites of reconnections between pulmonary veins (PVs) and left atrium in the acute phase (A, B, C) and in redo‐procedures (D, E, F). In the acute phase, five nonisolated PVs were identified (two right superior PVs, two right inferior PVs and one left superior PV). In all four redo‐procedures, reconnected PVs were identified (2 left superior PVs, 1 right inferior PV, 2 right superior PVs).

Procedure time was significantly longer in the EAM group, as expected (86.5 ± 11.4 vs. 78.4 ± 9.3 min, *p* .008), whereas no difference was reported in the fluoroscopy time (14.5 ± 4.8 vs. 13.5 ± 4.4 min, *p* .41). EAM identified 5 non isolated PVs (6.3%) that required the delivery of additional lesions (two right superior PVs, two right inferior PVs and one left superior PV). Successful final isolation was confirmed with remapping. In the EAM group, there was one case with common PV ostium (left PVs). No major or minor complications occurred in the cohort. Procedural characteristics are summarized in Table 2. Figure 1 shows procedural images from different patients of the cohort. Figure 2A–C shows schematically the sites of reconnections in the acute phase.

**TABLE 2:** Procedural characteristics and follow‐up.

	Total patients, *n* = 51	EAM group, *n* = 20	Fluoro‐only group, *n* = 31	*p*‐value
Fluoroscopy time (min)	13.8 ± 4.6	14.5 ± 4.8	13.4 ± 4.4	.41
Procedure time (min)	81.6 ± 10.8	86.5 ± 11.4	78.4 ± 9.3	.008
Number of nonisolated pulmonary veins		5/79 (6.3%)		
Common ostium	1	1 (LPVs)		
ATA recurrence rates	9 (17.6%)	4 (20%)	5 (16.1%)	.72

### 3.2 Arrhythmia recurrences during the follow‐up period and redo procedures

During a mean follow‐up period of 11.2 ± 1.3 months (blanking period of 8 weeks), 4 patients (20%) in the EAM group and 5 patients (16.1%) in the Fluoro‐only group had a documented ATA episode. The difference did not reach statistical significance (*p* .72). Four patients of the cohort underwent a redo‐procedure (3 in the EAM and 1 in the Fluoro‐only group). In all redo cases, reconnected PVs were identified (2 left superior PVs, 1 right inferior PV, 2 right superior PVs). Figure 2D–F shows the reconnected sites in the redo‐procedures. One patient received additionally a cavotricuspid isthmus (CTI) line because of documented CTI‐dependent atrial flutter.

## 4 DISCUSSION

Single‐shot ablation devices have accelerated procedure time for PVI, with the inherent limitation of being less versatile to possible anatomical variations. Most of these devices rely only to fluoroscopy for lesion guidance while the use of an EAM system is not mandated. As a consequence, even though successful PVI can be validated with electrophysiological maneuvers, operators are less aware of the extend and the localization of the delivered lesions at the PV ostium. The FARAPULSE™ PFA System (Boston Scientific, Natick, MA, USA) consists of a 12 F over‐the‐wire multipolar ablation catheter that is introduced to the left atrium through a dedicated 13 F steerable sheath. The ablation catheter has 20 electrodes that are equally distributed in five splines. The form of the splines can be modified in two major configurations, the basket and the flower form, designed to achieve circumferential PV antral isolation. The system includes a generator that produces high electric‐field pulses of short duration. PFA is delivered through all catheter electrodes in a bipolar fashion.^3^

The aim of our study was to assess whether the addition of EAM to validate PVI, previously achieved with the pentaspline PFA catheter, can enhance the efficacy of the technique. In our limited cohort of 51 patients, the addition of EAM did not affect the long‐term ATA recurrences. This may be explained by both the small sample size and the low rate of nonisolated PVs (5/79) that were identified acutely. A technical issue that should not be neglected is the reversible injury that PFA may induce in adjacent myocardial areas. These areas of stunned myocardium may falsely be identified as low voltage areas in voltage mapping in the acute phase and eventually recover in the follow‐up period.^1^ Dedicated studies are needed to investigate the accuracy of voltage mapping acutely when using PFA technology.

A limited number of studies in the literature have provided data from EAM in patients treated with this technique.^4^, ^5^, ^6^, ^7^, ^8^ An ablation protocol of 8 applications (four in basket and four in flower configuration) has been related to insufficient isolation in the area of left anterior antral PV segment, when validated acutely with EAM.^4^ To overcome this issue, in the protocol of our study we chose to include two extra applications in the flower configuration for each PV (four in basket and six in flower in total) aiming to the ablation of the anterior area by properly manipulating the catheter (anterior torque). This decision may have contributed to the high rate of successful acute isolation that we experienced.

The major endpoint of efficacy of all ablation techniques is the creation of durable lesions in the long‐term. A study that compared the results of PVI using the PFA catheter with cases of PVI using various forms of thermal energy suggested the creation of equally durable lesions with the novel technology (remap 75 days after the procedure was used to validate the results).^7^ In another study with cases of redo‐procedures after PFA, the rate of PV reconnection 6.1 ± 4 months after the initial procedure was reported to be as low as 9.1%, whereas the majority of macro‐reentrant atrial tachycardia manifestations were related to critical isthmuses at the posterior wall.^8^ Our study was not designed to assess long‐term durability of lesions. However, in the small number of our redo cases, at least one reconnected PV was identified. No cases of atrial tachycardias related to the PVI were found.

## 5 LIMITATIONS

The main limitations of our study are the small number of participants and the lack of randomization. Follow‐up with Holter monitoring was rigorous (1, 3, 6, 9, and 12 months). However, Holter monitoring may underestimate recurrence rates compared to long‐term monitoring devices.

## 6 CONCLUSION

In a cohort of patients with AF undergoing first time PVI using a multi‐electrode pentaspline PFA catheter, PVI validation with EAM did not lead to significantly different arrhythmia recurrence rates compared to PVI without EAM. In the acute phase, the rate of nonisolated PVs was low.

## FUNDING INFORMATION

None.

## CONFLICT OF INTEREST STATEMENT

The authors declare no conflicts of interests.

## CONSENT

Patients provided written informed consent for participation in the study.

## Data Availability

Data are available upon request to the corresponding author.

## References

[1] Wittkampf FHM , van Es R , Neven K . Electroporation and its relevance for cardiac catheter ablation. JACC Clin Electrophysiol. 2018;4(8):977–986. 10.1016/j.jacep.2018.06.005 30139498

[2] Turagam MK , Neuzil P , Schmidt B , Reichlin T , Neven K , Metzner A , et al. Safety and effectiveness of pulsed field ablation to treat atrial fibrillation: one‐year outcomes from the MANIFEST‐PF registry. Circulation. 2023;148(1):35–46. 10.1161/CIRCULATIONAHA.123.064959 37199171

[3] Badertscher P , Knecht S , Rosso R , Krisai P , Spreen D , Katic J , et al. How to perform pulmonary vein isolation using a Pentaspline pulsed field ablation system for the treatment of atrial fibrillation. Heart Rhythm. 2024;2:S1547–46. 10.1016/j.hrthm.2024.06.058 38964447

[4] Bohnen M , Weber R , Minners J , Jadidi A , Eichenlaub M , Neumann FJ , et al. Characterization of circumferential antral pulmonary vein isolation areas resulting from pulsed‐field catheter ablation. Europace. 2023;25(1):65–73. 10.1093/europace/euac111 35852306 PMC10103571

[5] Kueffer T , Baldinger SH , Servatius H , Madaffari A , Seiler J , Mühl A , et al. Validation of a multipolar pulsed‐field ablation catheter for endpoint assessment in pulmonary vein isolation procedures. Europace. 2022;24(8):1248–1255. 10.1093/europace/euac044 35699395

[6] Kawamura I , Neuzil P , Shivamurthy P , Petru J , Funasako M , Minami K , et al. Does pulsed field ablation regress over time? A quantitative temporal analysis of pulmonary vein isolation. Heart Rhythm. 2021;18(6):878–884. 10.1016/j.hrthm.2021.02.020 33647464

[7] Kawamura I , Neuzil P , Shivamurthy P , Kuroki K , Lam J , Musikantow D , et al. How does the level of pulmonary venous isolation compare between pulsed field ablation and thermal energy ablation (radiofrequency, cryo, or laser)? Europace. 2021;23(11):1757–1766. 10.1093/europace/euab150 34151947 PMC8576283

[8] Tohoku S , Chun KRJ , Bordignon S , Chen S , Schaack D , Urbanek L , et al. Findings from repeat ablation using high‐density mapping after pulmonary vein isolation with pulsed field ablation. Europace. 2023;25(2):433–440. 10.1093/europace/euac211 36427201 PMC9935020

